# miR-23a-3p as a Biomarker Associated with Prediabetes in People Living with HIV: An Integrative Analysis of Inflammatory, Metabolic, and Insulin Resistance Signatures

**DOI:** 10.3390/ijms27135658

**Published:** 2026-06-23

**Authors:** Paula Catalina Méndez-Ríos, Yusnier Lázaro Díaz-Rodríguez, Luis F. Jave-Suarez, Luz A. González-Hernández, Jaime F. Andrade-Villanueva, Monserrat Álvarez-Zavala, Pedro Martínez-Ayala, Vida V. Ruiz-Herrera, Elsa Janneth Anaya-Ambriz, Karina Sánchez-Reyes

**Affiliations:** 1 Universidad de Guadalajara, Centro Universitario de Ciencias de La Salud, Programa de Doctorado en Ciencias en Biología Molecular en Medicina, Guadalajara 44340, Mexico; catalina.mendez4084@alumnos.udg.mx; 2 Universidad de Guadalajara, Centro Universitario de Ciencias de La Salud, Programa de Maestría en Microbiología Médica, Guadalajara 44340, Mexico; yusnier.diaz9426@alumnos.udg.mx; 3 Instituto Mexicano del Seguro Social, Centro de Investigación Biomédica de Occidente, (CIBO-IMSS), División de Inmunología, Guadalajara 44340, Mexico; lfjave@gmail.com; 4 Universidad de Guadalajara, Centro Universitario de Ciencias de La Salud, Departamento de Clínicas Médicas, Instituto de Investigación en Inmunodeficiencias y VIH (InIVIH), Guadalajara 44350, Mexico; luceroga08@gmail.com (L.A.G.-H.); drjandradev@gmail.com (J.F.A.-V.); montserrat.zavala@academicos.udg.mx (M.Á.-Z.); 5 Hospital Civil de Guadalajara “Fray Antonio Alcalde”, Unidad de VIH, Guadalajara 44350, Mexico; pedro.martinez@cucs.udg.mx (P.M.-A.); vida.ruiz@academicos.udg.mx (V.V.R.-H.); 6 Universidad de Guadalajara, Centro Universitario de Ciencias de La Salud, Programa de Doctorado en Microbiología Médica, Guadalajara 44340, Mexico

**Keywords:** people living with HIV (PLWHIV), type 2 diabetes (T2D), prediabetes (preT2D), miR-23a-3p, chronic inflammation, insulin resistance (IR), biomarkers

## Abstract

People living with HIV (PLWHIV) have an increased risk of developing metabolic disorders, including type 2 diabetes (T2D), partly driven by chronic low-grade inflammation and immune dysregulation. This study evaluated the potential role of circulating miR-23a-3p as a possible early biomarker of prediabetes (preT2D) in PLWHIV. In this cross-sectional study, 80 adults were divided into five groups (*n* = 16 each): normoglycemic PLWHIV, PLWHIV with preT2D, PLWHIV with T2D, HIV-negative individuals with T2D, and controls. Clinical, anthropometric, biochemical, inflammatory, and insulin resistance (IR) markers were assessed, while plasma miR-23a-3p was quantified by digital PCR (dPCR). Bioinformatic network analysis was performed to identify potential molecular targets. PLWHIV with T2D showed the most unfavorable metabolic and inflammatory profile, including higher HbA1c, triglycerides, IL-6, TNF-α, hs-CRP, and GDF-15. In contrast, PLWHIV with preT2D exhibited significant overexpression of miR-23a-3p, whereas lower levels were observed in normoglycemic PLWHIV. miR-23a-3p correlated positively with IL-6 and GDF-15. ROC analyses showed good discriminative performance of miR-23a-3p for preT2D in PLWHIV (AUC = 0.80), and logistic regression confirmed its association with preT2D. In silico network analysis suggested potential inflammatory and metabolic targets of miR-23a-3p; however, these findings require experimental validation. These findings suggest that miR-23a-3p may represent a potential early biomarker of preT2D and immunometabolic dysfunction in PLWHIV.

## 1. Introduction

Human immunodeficiency virus (HIV) infection remains a major global public health concern. This virus is characterized by its ability to infect CD4^+^ T lymphocytes, leading to their progressive depletion and subsequent immunosuppression [1]. Although the implementation of antiretroviral therapy (ART) has significantly reduced mortality and increased the life expectancy of people living with HIV (PLWHIV), it has also contributed to a higher prevalence of non-communicable diseases (NCDs), including cardiovascular disease (CVD), type 2 diabetes (T2D), and liver and kidney diseases [2].

In PLWHIV, the prevalence of T2D has been estimated to be up to four times higher compared to the general population [3]. This increase cannot be explained solely by traditional risk factors but rather reflects the convergence of multiple complex pathophysiological mechanisms associated with the development of glucose metabolism alterations in PLWHIV.

The interaction between viral infection, prolonged exposure to ART, and chronic low-grade inflammation contributes to the progressive impairment of insulin sensitivity and promotes the development of dysglycemia [4]. This is partly related to the inhibition of GLUT4 translocation in muscle and adipose tissue [5].

At the molecular level, HIV infection induces a state of persistent immune activation characterized by the presence of viral proteins such as Nef and Tat, which activate intracellular pro-inflammatory pathways, including NF-κB and JAK/STAT. Consequently, a sustained inflammatory cascade is generated, driven by the continuous production of pro-inflammatory cytokines, even under conditions of viral suppression. Additionally, these viral proteins have been associated with the development of insulin resistance (IR) through alterations in adipogenesis [6,7].

Cytokines such as IL-6, TNF-α, and IL-8 play a central role in this inflammatory process, promoting IR through serine phosphorylation of IRS-1, inhibition of the PI3K/AKT pathway, and disruption of GLUT4-mediated glucose transport. In contrast, anti-inflammatory cytokines such as IL-10 may be reduced or functionally impaired, perpetuating an immunometabolic imbalance [8,9].

Concomitantly, in PLWHIV undergoing ART who develop T2D, increased inflammation and enhanced metabolic activity of CD4^+^ T cells have been associated with higher mitochondrial density and reduced oxygen consumption rates. This leads to increased production of reactive oxygen species (ROS), oxidative stress, and mitochondrial dysfunction, ultimately contributing to cellular damage and disruption of multiple metabolic pathways [10].

Growth Differentiation Factor 15 (GDF-15) is a soluble protein belonging to the TGF-β (Transforming Growth Factor beta) superfamily. It is considered a cellular and metabolic stress cytokine because its expression increases under conditions of tissue damage, inflammation, hypoxia, mitochondrial dysfunction, and aging [11,12]. It has also been involved in the regulation of lipid and glucose metabolism, reflecting cellular adaptations to mitochondrial dysfunction and impaired energy homeostasis [13].

The interplay between chronic inflammation, oxidative stress, and mitochondrial dysfunction establishes a pathophysiological axis that promotes the progressive development of IR and dysglycemia. However, these alterations occur subclinically during the early stages, limiting their detection when using conventional diagnostic tools.

In this context, surrogate indices derived from clinical parameters have emerged as useful tools to indirectly estimate these processes. Indices such as monocyte-to-lymphocyte ratio (MLR), Systemic Inflammation Response Index (SIRI), and Aggregate Index of Systemic Inflammation (AISI) reflect systemic inflammatory status by integrating cellular components of both innate and adaptive immunity as well as platelets [14].

Meanwhile, surrogate indices of IR, such as the triglyceride-glucose (TyG) index, Metabolic Score for IR (METS-IR), and Homeostatic Model Assessment for IR (HOMA-IR), combine biochemical, anthropometric, and glycemic variables to identify early alterations associated with IR, a key pathogenic mechanism preceding dysglycemia and T2D [15,16,17]. However, although these surrogate markers provide relevant systemic insights, they do not fully capture the complexity of the molecular mechanism underlying metabolic and inflammatory dysregulation. Increasing evidence suggests that many of the inflammatory and metabolic pathways are regulated at the post-transcriptional level by microRNAs (miRNAs) [18].

miRNAs have emerged as key regulators of immunometabolic homeostasis, as they can modulate multiple biological pathways simultaneously through the regulation of target genes involved in inflammatory responses, glucose metabolism, adipose tissue dysfunction, insulin signaling, and mitochondrial stress. Thus, they act as central nodes in the inflammation–metabolism axis, highlighting their potential as promising diagnostic tools due to their key role in epigenetic regulation [19,20,21].

In particular, miR-23a-3p has emerged as a potential regulator of inflammatory and metabolic processes, participating in the modulation of pathways associated with apoptosis, immune response, and energy homeostasis, positioning it as a possible integrative node in the pathophysiology of glycemic alterations [22,23,24].

However, despite its biological relevance, the role of miR-23a-3p in the early detection of glycemic alterations in PLWHIV has not yet been characterized. In this context, the integration of molecular biomarkers and clinical indices represents a promising strategy to comprehensively understand the mechanisms underlying the progression to overt diabetes in PLWHIV. Therefore, the aim of this study was to evaluate the potential of miR-23a-3p as a potential early biomarker of dysglycemia in PLWHIV, as well as to analyze its association with surrogate indices of inflammation and IR, and to explore its potential biological relevance through an integrative approach.

## 2. Results

### Clinical, Sociodemographic, and Anthropometric Characteristics

Sociodemographic and anthropometric data of the study group are summarized in Table 1. Significant age differences were observed between groups (*p* = 0.008), with the T2D group being the oldest (53 ± 9 years) compared to the normoglycemic PLWHIV group (38 ± 11 years). Among the PLWHIV groups, a predominance of males over females was observed (*p* = 0.006), whereas in the T2D and control groups, sex distribution was more homogeneous (T2D: 56.3% male vs. 43.7% female; Control: 50% male vs. 50% female).

Regarding the anthropometric measures, the PLWHIV+T2D group showed higher body weight compared with the normoglycemic PLWHIV group (84.9 ± 17 vs. 69.3 ± 9 kg, respectively; *p* = 0.035). Likewise, significant differences in BMI were observed between groups (*p* = 0.009), with higher medians in the T2D (29.4 kg/m^2^) and PLWHIV+T2D (28.4 kg/m^2^) groups. This pattern was consistent with that observed for the waist-to-hip ratio (*p* = 0.010).

Additionally, clinical characteristics related to HIV infection were analyzed (Table 2). A longer duration of infection was observed in the PLWHIV+preT2D group ($\bar{x}$ = 12 years), compared with the PLWHIV+T2D ($\bar{x}$ = 10 years) and normoglycemic PLWHIV ($\bar{x}$ = 6 years) groups. Consistently, ART duration showed differences between groups (*p* = 0.011). Interestingly, the groups with dysglycemia had been receiving ART for approximately 10 years, compared with 6 years among normoglycemic PLWHIV. In relation to viral load, all participants exhibited an undetectable viral load and CD4^+^ T cell counts above 400 cells/µL, indicating adequate immune reconstitution. The lowest nadir CD4^+^ T cells were observed in the PLWHIV+preT2D group, which, in turn, exhibited the highest CD4^+^/CD8^+^ ratio, with a value of 0.623, indicating that although they experienced severe immunosuppression, they responded favorably to treatment, and the metabolic dysfunction could be associated with accumulated immunometabolic damage, residual inflammation, and mitochondrial dysfunction.

Regarding the ART regimen, Biktarvy was the predominant regimen in all study groups. Two patients were receiving Atripla and Truvada + ATV/r, distributed in the PLWHIV+preT2D and PLWHIV+T2D groups, respectively. No statistically significant differences in ART regimens were observed between groups (*p* = 0.171).

Hematological, glycemic, and lipid profiles were evaluated (Table 3). Interestingly, a decrease in hemoglobin levels was found in the T2D group (13.5 ± 1.7 g/dL; *p* < 0.0001). Although hemoglobin values remained within the normal range, they were closer to the lower reference limit. Additionally, the percentage of monocytes was significantly lower in the T2D group (5.9 ± 2.5) and higher in normoglycemic PLWHIV (8.9 ± 2.2) (*p* = 0.012). The remaining evaluated parameters were within normal ranges, with no statistically significant differences observed between groups. 

In the glycemic profile, the expected alterations were observed according to each study group. Glucose levels were significantly higher in the T2D group (148 mg/dL (79–254)), followed by the PLWHIV+T2D group (138 mg/dL (80–281)) (*p* < 0.001). This trend was consistent with HbA1c levels, which showed a mean of 8.3% in PLWHIV+T2D, whereas normoglycemic PLWHIV had the lowest mean (5.4%) among the five study groups (*p* < 0.001). Likewise, insulin levels were higher in the groups with established glycemic alteration (*p* = 0.008), suggesting the presence of insulin resistance.

On the other hand, uric acid showed significant differences (*p* = 0.002), with elevated levels in the PLWHIV+T2D group (6 ± 1.4 mg/dL). In contrast, dyslipidemia was evidenced by significantly lower HDL-C levels (32 mg/dL (26–45)) and elevated triglyceride concentrations (180 mg/dL (76–546)) in the PLWHIV + T2D group, which could suggest an atherogenic profile in this group. Regarding the lipid profile, no significant differences were observed in total cholesterol, LDL-C, or VLDL-C.

Additionally, cytokines, immunometabolic markers, and surrogate indices of IR and inflammation were evaluated in the study groups to complement the characterization of systemic status (Table 4). Regarding the inflammatory profile, no statistically significant differences were observed in IL-6 levels; however, levels were higher in the PLWHIV+T2D group (2.9 pg/mL (0.68–16.7)). Likewise, no differences between groups were observed for IL-8 (*p* > 0.05).

Interestingly, IL-10 levels showed significant differences between groups (*p* = 0.017), with the PLWHIV+preT2D group presenting the lowest levels (1.6 pg/mL (0.7–7.9)), whereas the PLWHIV+T2D group exhibited the highest levels of this immunosuppressive cytokine (3 pg/mL (0.68–381)).

Given the central role of inflammatory and metabolic stress-related biomarkers in immunometabolic dysregulation, circulating levels of hs-CRP, TNF-α, and GDF-15 were found to be significantly increased in the PLWHIV+T2D group (18 mg/L (2–170)), *p* = 0.008; 41.2 pg/mL (29–54.7), *p* = 0.0074; and 729 pg/mL (437–1510), *p* < 0.0001, respectively). In contrast, the groups displaying the lowest median levels of these biomarkers were PLWHIV+preT2D (1.8 mg/L (0.7–150)) for hs-CRP, and the control group (30.2 pg/mL (23.5–47.9)) and (340 pg/mL (216–1033)) for TNF-α and GDF-15, respectively. Notably, the levels of the latter biomarker exhibited a progressively increasing trend as glycemic dysregulation advanced.

Given that chronic low-grade inflammation plays a central role in the progression of IR and cardiometabolic alterations, cell-derived inflammatory indices were evaluated. SIRI and AISI showed no significant differences between groups (*p* > 0.05), whereas the MLR index was significantly higher in PLWHIV+preT2D (0.018 ± 0.010; *p* = 0.0325), suggesting early activation at this stage of glycemic alteration.

To comprehensively assess glucose homeostasis and insulin metabolism, the indices HOMA-IR, HOMA-%S, and HOMA-%β were included, which enabled the estimation of IR, insulin sensitivity, and β-cell function, respectively. Statistically significant differences were identified across all surrogate indices of IR. Increased IR, reflected by higher HOMA-IR, TyG, and METS-IR values, concomitant with reduced insulin sensitivity (HOMA-%S), was observed in the groups with established T2D. Notably, among PLWHIV, both insulin sensitivity and β-cell function were markedly impaired following the onset of T2D, with median values of 38% (19–118) for HOMA-%S and 60% (10.4–274) for HOMA2-%β, respectively.

**Figure 1:**
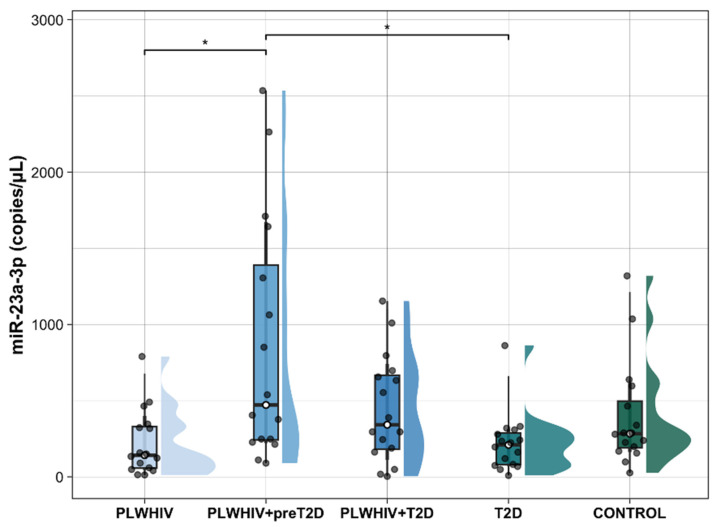
Plasma miR-23a-3p expression levels (copies/µL). Data are presented as half-violin plots combined with boxplots. The violin plot illustrates the distribution and density of the observations, the box represents the interquartile range (IQR), and the white circle and horizontal black line denote the median value of each group; the whiskers extend to 1.5 × IQR from the hinges. Individual points represent values from each participant. Group comparisons were performed using the Kruskal–Wallis test followed by Dunn’s multiple-comparison post hoc test. * *p* < 0.05. PLWHIV, people living with HIV; PLWHIV+preT2D, people living with HIV + prediabetes; PLWHIV+T2D, people living with HIV + diabetes; T2D, type 2 diabetes.

In this context and considering the regulatory role of miRNAs in the interaction between inflammation and metabolism, plasma expression levels of miR-23a-3p were analyzed (Figure 1). This miRNA showed wide variability among the analyzed groups. Notably, it was overexpressed in PLWHIV+preT2D with high interindividual variability (median: 471.9 (90.1–2535) copies/µL), being significantly higher compared with normoglycemic PLWHIV (134.2 (12–789) copies/µL) and the T2D group (median: 210.2 (9–861) copies/µL) (*p* = 0.0066).

In contrast, in the PLWHIV+T2D group (median: 342.3 (3.5–1154) copies/µL) and the control group (median: 284 (26–1319) copies/µL), miR-23a-3p lower expression was observed, without statistically significant differences compared with the other study groups. These findings demonstrate an overexpression of this miRNA associated with early stages of glycemic alterations in PLWHIV.

**Figure 2:**
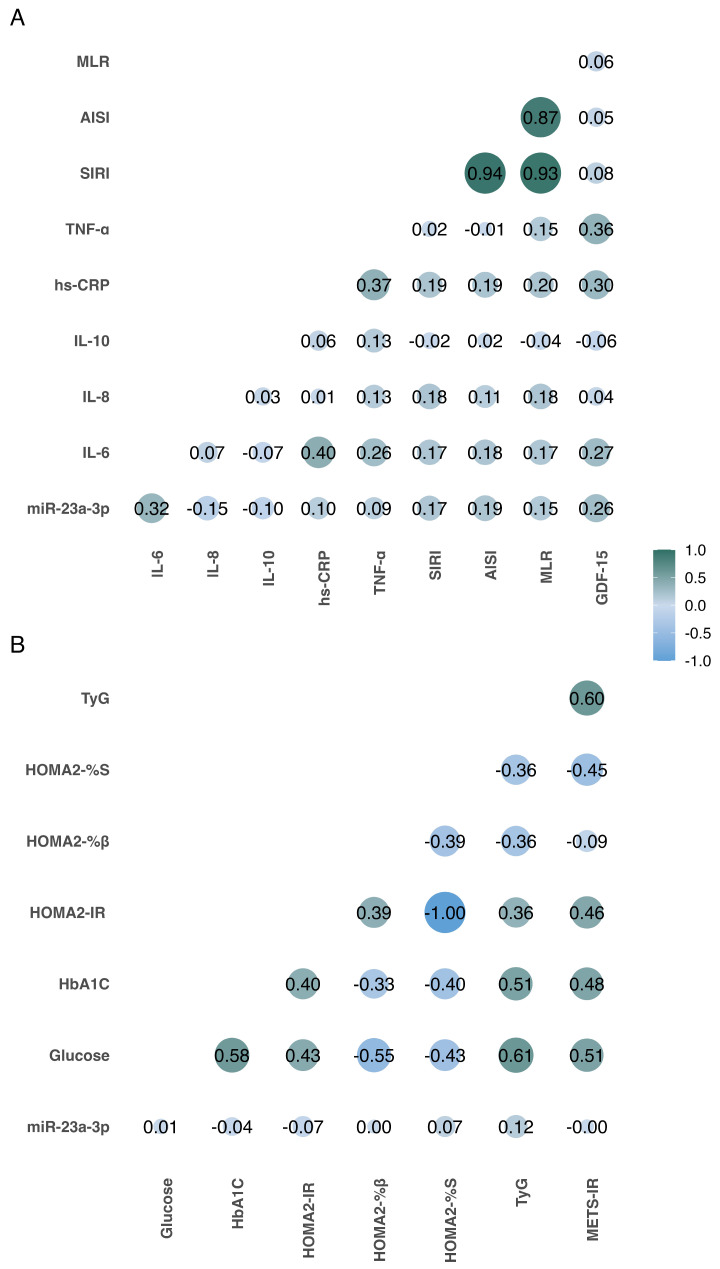
Correlation matrix of miR-23a-3p: (**A**) Inflammatory, systemic inflammation-related and metabolic stress biomarkers; (**B**) glycemic and surrogate insulin resistance indices. The color and size of the circles represent the direction and magnitude of the correlation, respectively, and the number corresponds to the Spearman correlation coefficient (ρ).

Considering this pattern of differential expression, the relationship between miR-23a-3p and metabolic and inflammatory markers was evaluated (Figure 2A), as well as with inflammation and insulin resistance indices (Figure 2B), to explore its possible role as a modulator in the interaction between chronic low-grade inflammation and metabolic alterations.

Regarding metabolic and inflammatory markers (Figure 2A), moderate and statistically significant positive correlations were observed between miR-23a-3p and IL-6 (ρ = 0.32, *p* = 0.004; q = 0.0241), and GDF-15 (ρ = 0.26, *p* = 0.020, q = 0.0835). In contrast, weak and non-significant positive correlations were identified with hs-CRP, TNF-α, AISI, SIRI, and MLR (hs-CRP: ρ = 0.10, *p* = 0.376, q = 0.589; TNF-α: ρ = 0.09, *p* = 0.402, q = 0.603; AISI: ρ = 0.19, *p* = 0.09, q = 0.28; SIRI: ρ = 0.17, *p* = 0.14, q = 0.30; MLR: ρ = 0.15, *p* = 0.18, q = 0.36). On the other hand, weak non-significant negative correlations were shown with IL-8 and IL-10 (IL-8: ρ = −0.15, *p* = 0.18, q = 0.36; IL-10 ρ = −0.10, *p* = 0.38, q = 0.59), suggesting that miR-23a-3p expression is not strongly associated with systemic inflammatory status in this cohort.

Regarding the metabolic profile (Figure 2B), miR-23a-3p expression was not associated with markers of glucose homeostasis, insulin resistance, or lipid metabolism. Correlation analyses revealed no significant relationships between miR-23a-3p and fasting glucose, HbA1c, METS-IR, HOMA2-IR, HOMA-2%β, HOMA2-%S, or the TyG index (all *p* > 0.05 and q > 0.05). These findings suggest that miR-23a-3p expression is largely independent of the metabolic status of the studied population.

Overall, these findings suggest that miR-23a-3p is preferentially associated with markers of the inflammatory axis, particularly mediators related to systemic inflammation and cellular stress, while showing no direct relationship with the metabolic parameters evaluated.

**Figure 3:**
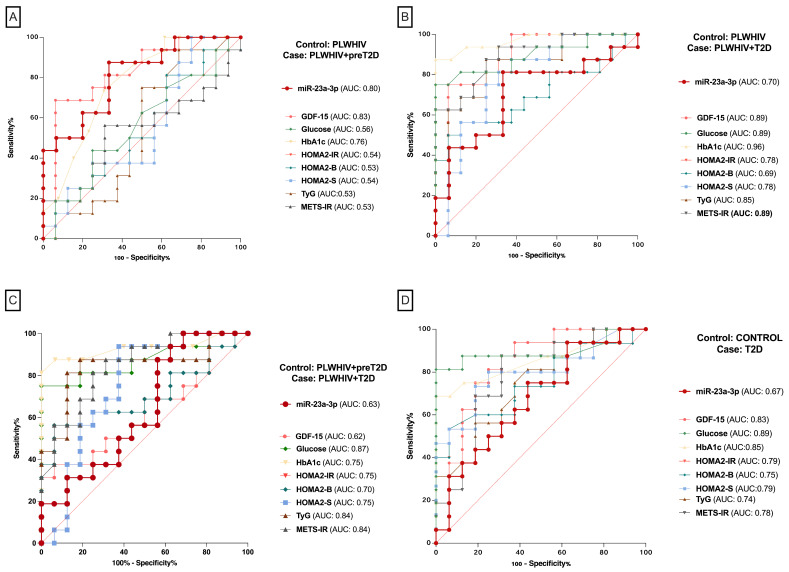
ROC curve analysis for the evaluation of the discriminatory capacity of biomarkers of interest across the different study groups. The discriminatory capacity of biomarkers was assessed using ROC curves in three clinical scenarios: (**A**) PLWHIV vs. PLWHIV+preT2D; (**B**) PLWHIV vs. PLWHIV+T2D; (**C**) PLWHIV+preT2D vs. PLWHIV+T2D; (**D**) Control vs. T2D. The diagonal line represents a non-discrimination line. miR-23a-3p showed better performance in early stages, whereas classical markers such as HbA1c and glucose stood out in advanced stages. PLWHIV, people living with HIV; PLWHIV+preT2D, people living with HIV + prediabetes; PLWHIV+T2D, people living with HIV + diabetes; T2D, type 2 diabetes.

Area under the curve (AUC) analyses were performed (Figure 3) to evaluate the discriminatory capacity of miR-23a-3p compared with metabolic and inflammatory biomarkers under clinical scenarios: PLWHIV vs. PLWHIV+preT2D (Figure 3A), PLWHIV vs. PLWHIV+T2D (Figure 3B), PLWHIV+preT2D vs. PLWHIV+T2D (Figure 3C), and Control vs. T2D (Figure 3D).

In the early glycemic alteration scenario (PLWHIV+preT2D), Figure 3A, miR-23a-3p showed the best diagnostic performance (AUC = 0.80), comparable to GDF-15 (AUC = 0.83) and was superior to the traditional biomarker HbA1c (AUC = 0.76), as well as METS-IR (AUC = 0.53). In contrast, HOMA-derived indices (HOMA2-IR, HOMA2-%β, and HOMA2-%S) and glucose displayed poor and limited discriminatory capacity, with AUC values of 0.54, 0.53, and 0.56, respectively. These results should be interpreted with caution, as potential confounding due to differences in age and ART duration between groups may have influenced the observed AUC values.

In Figure 3B (PLWHIV+T2D), classical glycemic biomarkers showed better diagnostic performance. HbA1c exhibited the highest discriminatory power (AUC = 0.96), followed by glucose (AUC = 0.89). It is important to highlight the discriminatory ability of the GDF-15 (AUC = 0.89) in this scenario. Surrogate IR indices showed moderate diagnostic performance: TyG (AUC = 0.85), HOMA2-IR (AUC = 0.78), and METS-IR (AUC = 0.89). In contrast to the early-stage scenario, miR-23a-3p showed only moderate performance in this group (AUC = 0.70), suggesting reduced discriminatory capacity in advanced glycemic dysregulation.

Otherwise, miR-23a-3p showed limited discrimination between PLWHIV with preT2D and PLWHIV with T2D (AUC = 0.63) (Figure 3C), compared with conventional metabolic markers, such as glucose (AUC = 0.87), TyG index (AUC = 0.84), and METS-IR (AUC = 0.84). The lower performance observed in this comparison suggests that miR-23a-3p alterations may occur predominantly during early dysglycemic stages rather than during progression to established T2D.

In Figure 3D (T2D), glucose showed the highest diagnostic performance (AUC = 0.89), followed by HbA1c (AUC = 0.85) and GDF-15 (AUC = 0.83). Surrogate IR indices demonstrated moderate discriminatory capacity, including HOMA2-IR (AUC = 0.79), METS-IR (AUC = 0.78), and TyG (AUC = 0.74). In contrast, miR-23a-3p displayed the lowest discriminatory performance in this setting (AUC = 0.67).

Overall, these results demonstrate a differential behavior of biomarkers across the spectrum of glycemic dysregulation. In early stages among PLWHIV, miR-23a-3p and GDF-15 exhibited superior diagnostic performance compared with traditional biomarkers, which displayed lower sensitivity during this phase. In contrast, in established T2D, classical markers such as HbA1c and glucose demonstrated the highest performance. Collectively, these results suggest that miR-23a-3p may represent a valuable potential biomarker for the early detection of glycemic alterations in PLWHIV, whereas GDF-15 appears to maintain consistent diagnostic performance throughout the progression of dysglucemia.

To translate these findings into a clinical context and establish the diagnostic value and clinical utility of the evaluated biomarkers, optimal cut-off points were determined using the Youden index, taking into account just the comparison with an AUC higher than or equal to 0.65 (Table 5). For each study group, only biomarkers exhibiting at least moderate predictive performance were included.

In early glycemic alteration (PLWHIV+preT2D), miR-23a-3p showed a cut-off point of >185.9 copies/µL, associated with high sensitivity (87.5%) and moderate specificity (66.6%). Meanwhile, GDF-15 showed a threshold of >590.6 pg/mL, characterized by high specificity (93.7%) but lower sensitivity (68.7%).

In PLWHIV+T2D, traditional biomarkers showed the best diagnostic performance, reaching 100% specificity. The optimal cut-off values were >6.1% for HbA1c and >103.5 mg/dL for glucose. In this context, GDF-15 demonstrated excellent diagnostic performance (AUC = 0.89), with a cut-off value of >581.5 pg/mL, yielding 75% sensitivity and 93% specificity. Similarly, the TyG index showed good discriminatory capacity (AUC = 0.85), with an optimal cut-off value of >8.745, reaching 87% sensitivity and 75% specificity. HOMA-IR showed moderate performance, with both sensitivity and specificity of 75% at a cut-off value of >1.66. Comparable findings were observed for METS-IR (AUC = 0.89), which showed a cut-off value of >45, with acceptable sensitivity (81%) and specificity (62%). Finally, in this clinical context, miR-23a-3p showed moderate discriminatory performance, with 81% sensitivity but a specificity below 70%.

Finally, in the comparison between T2D and the control group, glucose (>101 mg/dL) and HbA1c (>6.4%) showed the highest diagnostic performance, both reaching 100% specificity, with sensitivities of 81% and 68%, respectively. Regarding insulin resistance surrogates, HOMA2-IR (>2.125) demonstrated a robust discriminatory capacity with an AUC of 0.79 (95% CI: 0.6268−0.9566; *p* = 0.0057), yielding a sensitivity of 80% and a specificity of 75%. This was followed by METS-IR (>42.2), which achieved an AUC of 0.78 (95% CI: 0.6170−0.9455; *p* = 0.0067) with 87% sensitivity and 68% specificity, and the TyG index (>9.185), which exhibited an AUC of 0.74 (95% CI: 0.5797−0.9163; *p* = 0.0167), alongside a sensitivity of 56% and a specificity of 81%. In contrast, GDF-15 (>393.5 pg/mL) showed a sensitivity of 87% and a specificity of 68%. Under this condition, miR-23a-3p (<279.7 copies/µL) showed the lowest discriminatory performance, characterized by reduced sensitivity (75%) and specificity (56%).

Overall, these findings reinforce the distinct behavior of biomarkers throughout dysglycemia progression. In the early stages among PLWHIV, miR-23a-3p was characterized by high sensitivity, highlighting its potential utility as an early detection biomarker. In contrast, during advanced stages of glycemic dysregulation, traditional biomarkers such as glucose and HbA1c demonstrated superior diagnostic performance, primarily driven by their higher specificity.

Considering the consistency of these findings, the association between miR-23a-3p levels and preT2D in PLWHIV was further explored using logistic regression models under different analytical approaches (Table 6), considering both its categorization based on the optimal cut-off point (>185.9 copies/µL) and its behavior as a continuous variable. Given the limited sample size (*n* = 32), the unadjusted categorical model was considered the primary analysis, whereas adjusted and continuous-variable models were interpreted as exploratory.

**Figure 4:**
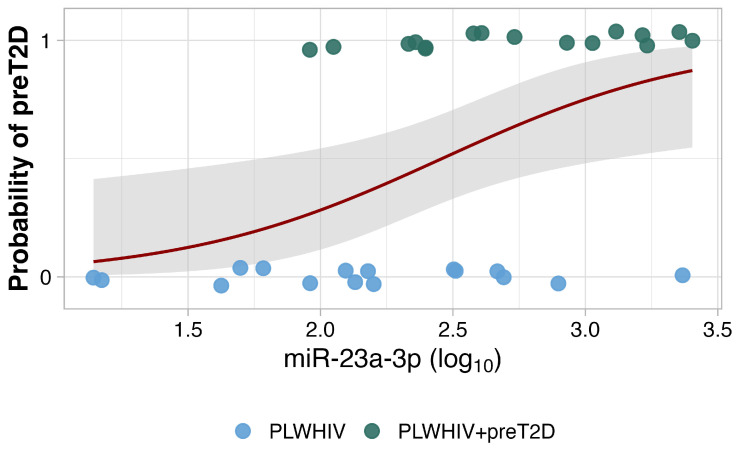
Predicted probability of preT2D according to circulating miR-23a-3p levels in PLWHIV. Individual observations are shown as scatter points for normoglycemic PLWHIV (blue) and PLWHIV with preT2D (green). The fitted logistic regression curve (red) illustrates the increase in the predicted probability of preT2D with higher miR-23a-3p expression levels (log_10_-transformed log_10_-transformed for visualization purposes). The shaded region represents the 95% confidence interval of the model prediction. PLWHIV, people living with HIV; PLWHIV+preT2D, people living with HIV + prediabetes.

As shown in Figure 4, higher miR-23a-3p expression levels were associated with an increased probability of preT2D. Individuals with miR-23a-3p levels above the optimal cut-off value (>185.9 copies/µL) showed significantly higher odds of preT2D compared with those below this threshold (OR = 7.22; 95% CI: 1.57–42.27; *p* = 0.0163). The logistic regression curve illustrates a progressive increase in the predicted probability of preT2D as miR-23a-3p expression increased, which is consistent with the discriminatory capacity observed in the ROC analysis. Although some overlap between groups was present, higher miR-23a-3p levels were predominantly observed among PLWHIV with preT2D (Figure 4).

To explore the robustness of this association, additional models were evaluated. After adjustment for age and BMI, the magnitude and direction of the association remained similar (OR = 4.99; 95% CI: 0.89–33.30; *p* = 0.074), although statistical significance was not maintained. Likewise, when miR-23a-3p expression was analyzed as a continuous variable (log10-transformed), a significant association was observed (OR = 14.67; 95% CI: 2.62–168.9; *p* = 0.0009). However, given the wide confidence intervals and limited number of participants, these adjusted analyses should be considered exploratory, requiring independent validation, and should be interpreted with caution.

Interestingly, these findings demonstrate a consistent cross-sectional association between plasma miR-23a-3p levels and preT2D in PLWHIV, reinforcing a significant relationship between increased miR-23a-3p expression and the presence of dysglycemia. Moreover, the logistic regression curve suggests a progressive increase in the probability of preT2D across higher miR-23a-3p expression levels.

To further investigate the molecular mechanisms potentially involved in the association between miR-23a-3p and early glycemic alterations in PLWHIV, an interaction network was constructed based on experimentally validated targets identified through functional assays and complemented with predictive in silico interactions (Supplementary Figure S1). This analysis was intended to explore the biological plausibility of the observed clinical associations and should therefore be regarded as hypothesis-generating rather than mechanistic evidence. Consequently, the identified targets and pathways require experimental validation in relevant biological models. The functional clustering visually segregates the targets into specific biological axes (Figure S1A), while the structural layout of the interactome highlights the individual protein conformations within a decentralized hub architecture (Figure S1B). Throughout the network, the strength of interactions was represented according to the level of available evidence, prioritizing targets with greater experimental support.

The network analysis showed that miR-23a-3p is highly connected with genes involved in inflammatory processes, immune response regulation, and intracellular signaling. Among the most interconnected nodes were *TNFAIP3*, *TRAF5*, *IRF1*, *IL6R*, and *IL8*, all of which have recognized roles in the modulation of inflammatory and immune pathways. Additional targets, including *PTEN*, *SPRY2*, and *PRKCA*, were associated with the regulation of cellular signaling and the integration of metabolic and inflammatory responses. In addition, we found a weak, non-significant negative correlation with IL-8 (ρ = −0.15, *p* = 0.18, q = 0.36).

To complement the topological architecture of the network and provide mathematical validation to these observations, an over-representation analysis was performed using the STRING Enrichment module. The statistical outcomes confirmed that these 25 target genes do not function in isolation but are over-represented in highly coordinated pathways with strict significance thresholds (Supplementary Table S1).

The bioinformatic enrichment robustly validated the immune–inflammatory axis, showing significant over-representation in GO terms such as response to cytokine (GO:0034097, *p* < 0.0001, FDR < 0.001) and cellular response to cytokine stimulus (GO:0071345, *p* < 0.0001, FDR < 0.01). This inflammatory profile was further supported by KEGG pathways, including the NF-kappa B signaling pathway (hsa04064, *p* < 0.001, FDR < 0.05) and TNF signaling pathway (hsa04668, *p* < 0.001, FDR < 0.05), as well as the IL-18 signaling pathway from WikiPathways (WP4754, *p* < 0.001, FDR < 0.05).

## 3. Discussion

In recent years, evidence regarding the risk faced by PLWHIV of developing metabolic diseases, particularly T2D, has increased, with a reported frequency of up to four times higher compared to the general population, occurring at younger ages and without direct association with obesity [25].

In this pathophysiological context, multiple factors interact in a complex manner. In this way, miR-23a-3p has been implicated as a possible modulator of inflammation and metabolism, participating in molecular mechanisms that involve the insulin receptor pathway, immune response, and oxidative stress. Current scientific literature reports alterations in its expression levels in contexts of dysglycemia and T2D, which positions it as a biomarker of metabolic dysfunction [23,26].

The demographic and clinical consistency between our cohort and established risk factors underscores a multifactorial etiology for dysglycemia in PLWHIV. Beyond traditional drivers like aging and visceral adiposity [15,27,28,29], the association with prolonged HIV and ART exposure highlights a cumulative immunometabolic toll. Chronic systemic inflammation and persistent immune activation likely accelerate metabolic decay. Furthermore, our alignment with data on NRTI-related T2D prevalence suggests that drug-induced mitochondrial toxicity and subcutaneous lipoatrophy remain critical, active pathways in long-term metabolic impairment [30].

Regarding immunovirological parameters, all PLWHIV participants were virologically suppressed and exhibited adequate immune reconstitution. Nevertheless, the PLWHIV+preT2D group presented the lowest nadir CD4^+^ T-cell counts despite showing the highest CD4^+^/CD8^+^ ratio, suggesting differences in previous immunological status among the study groups. This observation supports the concept that immunometabolic risk in PLWHIV may be influenced not only by current virological control but also by the cumulative biological impact of prior immune dysfunction.

Despite virological suppression and quantitative immune reconstitution, the lower nadir CD4^+^ count observed in the PLWHIV+preT2D group may reflect a history of more profound immune depletion before ART initiation. Previous studies have suggested that severe immunosuppression can be associated with persistent immune activation, residual inflammation, and long-term metabolic alterations despite successful viral suppression. Therefore, the lower nadir CD4^+^ count observed in the prediabetic group may indicate that historical immune damage contributes to the immunometabolic disturbances associated with dysglycemia in PLWHIV.

The metabolic profile observed in the PLWHIV+T2D group is consistent with a state of advanced IR accompanied by impaired β-cell function, as reflected by the elevated TyG, METS-IR, and HOMA2-IR indices. Beyond the expected alterations in glycemic markers, the coexistence of hypertriglyceridemia, elevated VLDL-C, and reduced HDL-C suggests a profound disturbance in lipid handling and energy metabolism [31,32,33,34]. This pattern has been associated with increased ectopic lipid deposition, adipose tissue dysfunction, and chronic low-grade inflammation, all of which contribute to the progression of metabolic disease in PLWHIV [35,36].

Importantly, HIV infection and long-term ART exposure may further amplify these alterations through mechanisms involving mitochondrial dysfunction, altered adipocyte biology, and persistent immune activation. Together, these processes may create a self-perpetuating immunometabolic environment in which inflammation, dyslipidemia, and IR reinforce one another, potentially accelerating progression from dysglycemia to overt T2D in PLWHIV.

A more pronounced inflammatory profile was observed in the PLWHIV+T2D group, characterized by higher IL-6, TNF-α, and hs-CRP concentrations. These findings are consistent with previous reports linking persistent immune activation and low-grade inflammation with IR and metabolic dysfunction in PLWHIV [9,37,38].

These inflammatory mediators have been implicated in the disruption of insulin signaling pathways and adipose tissue homeostasis, thereby contributing to the development and maintenance of IR. In addition, elevated hs-CRP concentrations support the presence of a persistent systemic inflammatory state, suggesting that chronic inflammation may represent a key biological link between HIV-associated immune dysregulation and metabolic deterioration [9,37,38].

Interestingly, IL-10 concentrations were also higher in the PLWHIV+T2D group. Although IL-10 is generally considered an anti-inflammatory cytokine, increased levels have been reported in settings of chronic immune activation, where pro- and anti-inflammatory mediators coexist. In this context, the concomitant elevation of IL-10 alongside IL-6, TNF-α, and hs-CRP may reflect an ongoing compensatory response aimed at limiting excessive inflammation rather than an effective resolution of the inflammatory process. This finding supports the concept that advanced metabolic dysfunction in PLWHIV occurs within a complex immunoregulatory environment characterized by the simultaneous activation of inflammatory and counterregulatory pathways [39,40,41].

Regarding the hematological inflammatory indices, the higher SIRI and MLR values observed in the PLWHIV+preT2D group suggest a relative predominance of myeloid cell populations, particularly monocytes and neutrophils, over lymphocyte-mediated responses. Because both indices incorporate monocyte counts as a central component, their elevation may reflect enhanced innate immune activation, a process that has been closely linked to persistent inflammation, microbial translocation, and metabolic disturbances in PLWHIV. Monocytes are recognized contributors to adipose tissue inflammation and insulin resistance through the production of pro-inflammatory mediators and the recruitment of additional immune cells to metabolically active tissues [14,15,16,17].

Interestingly, the predominance of these indices in the prediabetic rather than the diabetic stage may suggest that innate immune activation is particularly relevant during the early phases of metabolic deterioration, potentially preceding more overt metabolic dysfunction. Conversely, the higher AISI values observed in normoglycemic PLWHIV indicate a distinct inflammatory pattern that incorporates platelet counts in addition to neutrophils, monocytes, and lymphocytes. Although the biological significance of this finding remains uncertain, it may reflect differences in vascular or platelet-related inflammatory processes that are not directly associated with dysglycemia. Given the limited evidence regarding the application of these indices in PLWHIV, further studies are needed to determine whether they may serve as accessible markers of early immunometabolic alterations in this population.

GDF-15 is an emerging integrative biomarker of cellular stress and metabolic deterioration whose expression increases in response to chronic inflammation, tissue injury, oxidative stress, and mitochondrial dysfunction. In the context of HIV infection, many of these processes may persist despite effective virological suppression. Therefore, the higher GDF-15 concentrations observed in PLWHIV with increasing degrees of glycemic impairment may reflect not only worsening metabolic status but also the cumulative burden of chronic immunometabolic stress. This interpretation is supported by the positive correlations observed between GDF-15 and the pro-inflammatory cytokines IL-6 and TNF-α, suggesting that metabolic and inflammatory pathways remain closely interconnected in PLWHIV. Thus, rather than functioning solely as a marker of inflammation, GDF-15 may represent a biological indicator of the convergence between persistent immune activation, metabolic dysfunction, and cellular stress, all of which are recognized contributors to HIV-associated comorbidities [11,42,43,44,45].

In contrast to the progressive increase observed for GDF-15, miR-23a-3p displayed a distinct expression pattern, reaching its highest levels in the PLWHIV+preT2D group and decreasing thereafter in individuals with established T2D. This divergent behavior suggests that these biomarkers may reflect different stages of the immunometabolic response. Whereas GDF-15 appears to increase in parallel with metabolic deterioration, miR-23a-3p may be more closely associated with biological processes occurring during the transition from normoglycemia to overt diabetes.

Interestingly, the overexpression of miR-23a-3p in the prediabetic stage was not accompanied by significant correlations with glucose, HbA1c, or IR indices. This finding suggests that circulating miR-23a-3p does not simply mirror conventional markers of glycemic impairment. Instead, its expression pattern may reflect regulatory mechanisms activated during the early phases of metabolic dysfunction before more severe metabolic alterations become established. Previous studies have shown that miR-23a-3p participates in the regulation of inflammatory signaling pathways, cellular stress responses, and insulin-related pathways, supporting a potential role in immunometabolic adaptation [21].

Although the correlations between miR-23a-3p and IL-6 or GDF-15 were modest, they provide additional insight into the biological context in which this miRNA may operate. IL-6 is a central mediator of chronic low-grade inflammation and has been implicated in the regulation of several miRNAs through JAK/STAT-dependent signaling pathways. Therefore, the observed association may not necessarily indicate a direct interaction but rather reflect the influence of a shared inflammatory environment [46,47]. Likewise, although direct mechanistic interactions between miR-23a-3p and GDF-15 have not been clearly established, both molecules may respond to common biological stimuli related to chronic inflammation, cellular stress, and immunometabolic dysfunction. Consequently, the correlation observed in the present study may reflect convergence within shared adaptive pathways rather than a direct regulatory relationship.

Interestingly, previous studies have reported expression patterns consistent with our findings. miR-23a-3p levels have been shown to decline in individuals with established T2D compared with those in prediabetic stages, while higher expression has been observed in subjects with impaired glucose tolerance who subsequently progressed toward T2D [22,48]. Collectively, these observations support the hypothesis that miR-23a-3p may be particularly relevant during the early stages of metabolic deterioration, when compensatory and regulatory mechanisms are still active.

Taken together, the observed pattern suggests that miR-23a-3p may represent a possible biomarker of early immunometabolic adaptation rather than a direct indicator of hyperglycemia itself. Within this framework, the overexpression observed in PLWHIV+preT2D could reflect an adaptive response to persistent inflammatory and metabolic stress that becomes attenuated as metabolic dysfunction progresses. However, given the cross-sectional design of the study and the modest strength of the observed correlations, this interpretation should be considered exploratory and requires confirmation in larger prospective cohorts.

However, alternative explanations should also be considered when interpreting these findings. Previous studies have shown that HIV infection and long-term ART exposure can alter host miRNA expression profiles through persistent immune activation, chronic inflammation, and metabolic disturbances [49]. Likewise, age-related changes in miR-23a-3p expression have been reported [50], while sex-related hormonal and genetic factors may also influence miRNA regulatory networks [51]. In addition, adiposity has been associated with lower miR-23a-3p expression and adverse metabolic parameters, including increased BMI, waist circumference, and HOMA-IR [23].

Nevertheless, these variables do not appear to fully account for the expression pattern observed in our cohort. The PLWHIV+preT2D group, which exhibited the highest miR-23a-3p expression levels, did not present the greatest age or adiposity burden, and showed clinical characteristics broadly comparable to those of the PLWHIV+T2D group with respect to HIV disease duration and cumulative ART exposure. Therefore, although these factors may have contributed to interindividual variability, they are unlikely to entirely explain the marked overexpression observed in the prediabetic group. This observation supports the possibility that miR-23a-3p may be associated with specific biological changes occurring during the prediabetic stage, although this hypothesis requires confirmation in larger prospective studies.

From a clinical perspective, the discriminative performance observed for miR-23a-3p suggests that this miRNA may have potential utility for identifying early dysglycemia in PLWHIV. However, because the optimal thresholds were derived and evaluated within the same cohort, their applicability beyond the present study remains uncertain and requires validation in larger independent populations before any clinical implementation can be considered.

Previous studies have reported a diagnostic performance for miR-23a comparable to that observed in our cohort, with an AUC of 0.835 for distinguishing individuals with T2D from normoglycemic controls [22]. Interestingly, lower AUC values have been described in more advanced complications such as diabetic nephropathy, suggesting that the diagnostic utility of miR-23a-3p may vary according to the biological stage or metabolic condition being evaluated [52]. In this context, the relatively strong performance observed in PLWHIV with preT2D supports the hypothesis that miR-23a-3p may be particularly informative during the early phases of metabolic deterioration.

The diagnostic profile of GDF-15 appears to reflect a different biological dimension. GDF-15 has consistently shown excellent diagnostic performance across a variety of metabolic disorders [11,43]. Meta-analytic evidence has reported AUC values exceeding 0.90 for the identification of IR in T2D, while additional studies have demonstrated utility for detecting diabetes-related complications such as renal impairment [53,54]. Therefore, the performance observed in the present study further supports the concept that GDF-15 captures the cumulative burden of immunometabolic dysfunction rather than isolated glycemic alterations.

Interestingly, the sensitivity and specificity profiles of both biomarkers suggest potentially complementary clinical roles. Whereas miR-23a-3p showed higher sensitivity for identifying PLWHIV with prediabetes, GDF-15 displayed greater specificity. This pattern raises the possibility that miR-23a-3p may be more useful for detecting early immunometabolic alterations, while GDF-15 may provide additional information regarding the severity or persistence of underlying biological stress. Although speculative, such complementarity could be particularly valuable in PLWHIV, a population in which metabolic dysfunction develops through the interaction of chronic inflammation, immune activation, aging, and traditional metabolic risk factors.

The association observed between miR-23a-3p expression and prediabetes was further supported by logistic regression analyses. Rather than serving as independent proof of diagnostic utility, these findings provide additional evidence that the relationship between miR-23a-3p and prediabetes is unlikely to be explained solely by random variation within the study population. Nevertheless, prospective studies will be necessary to determine whether this miRNA possesses true predictive value for the future development of T2D in PLWHIV.

Network analysis identified several validated and predicted targets of miR-23a-3p involved in immune regulation, inflammatory signaling, and metabolic homeostasis, including *TNFAIP3*, *IL6R*, *PTEN*, *TRAF5*, *IRF1*, and *SPRY2*. Although these findings do not establish a direct mechanistic link with the clinical observations reported here, they provide a biologically plausible framework through which miR-23a-3p may influence immunometabolic processes. Notably, some of the identified targets are involved in pathways closely related to the alterations observed in our cohort, including IL6R, which participates in IL-6-mediated inflammatory signaling, *PTEN*, a key regulator of insulin signaling pathways, and *TNFAIP3*, an important modulator of NF-κB-dependent inflammatory responses.

Nevertheless, these results were derived exclusively from in silico analyses based on publicly available databases, and neither the predicted targets nor the enriched pathways were experimentally evaluated in the present cohort. Therefore, the proposed interactions should be considered exploratory and hypothesis-generating rather than evidence of causality. Future studies incorporating transcriptomic, proteomic, and functional validation approaches will be necessary to determine whether these molecular pathways contribute to the immunometabolic alterations observed in PLWHIV with dysglycemia.

Among the main limitations of the present study are the relatively small sample size, which may have influenced the precision of the estimates and limited the generalizability of the findings, and the cross-sectional design, which precludes the establishment of causal relationships. Consequently, it cannot be determined whether the observed alterations in miR-23a-3p expression precede, accompany, or result from dysglycemia. Furthermore, because the study groups consisted of different individuals rather than longitudinally followed participants, the differences observed between glycemic categories should not be interpreted as evidence of disease progression. Instead, they may also reflect the influence of factors such as age, duration of HIV infection, cumulative ART exposure, genetic susceptibility, or other unmeasured clinical variables.

Despite these limitations, this study provides novel evidence linking miR-23a-3p expression with HIV-associated prediabetes. Notably, the expression pattern observed across glycemic stages, together with its associations with inflammatory and stress-related biomarkers, suggests that miR-23a-3p likely reflects early immunometabolic adaptations rather than simply mirroring conventional measures of glycemic control. This interpretation is further supported by the distinct behavior of miR-23a-3p compared with GDF-15, highlighting the possibility that these biomarkers capture different aspects of the immunometabolic disturbances associated with HIV infection.

Among the strengths of this study are the inclusion of well-characterized groups representing different glycemic conditions within the context of HIV infection, the comprehensive evaluation of inflammatory, metabolic, and stress-related biomarkers, and the enrollment of exclusively virologically suppressed PLWHIV, minimizing the confounding effect of active viral replication. In addition, the integration of correlation, ROC, logistic regression, network topology, and functional enrichment analyses provided a multidimensional assessment of the potential role of miR-23a-3p in HIV-associated prediabetes.

Overall, our findings support the hypothesis that miR-23a-3p is a biomarker associated with prediabetes in PLWHIV. Nevertheless, larger prospective studies incorporating longitudinal follow-up and functional validation approaches will be necessary to determine whether miR-23a-3p possesses predictive value for progression to T2D and to further elucidate its biological role in the complex interplay between chronic inflammation, metabolic dysfunction, and HIV infection.

## 4. Materials and Methods

### 4.1. Study Population and Ethical Considerations

A cross-sectional, analytical, and descriptive study was conducted at the Institute of Immunodeficiencies and HIV (InIVIH) of the University of Guadalajara, in Guadalajara, Jalisco, Mexico. This study was carried out in accordance with the ethical principles of the Declaration of Helsinki (revised in October 2024) [55]. 

### 4.2. Participant Selection

A non-probabilistic, intentional, and matched sampling method was used. A total of 80 adult participants were included. General inclusion criteria considered adults of both sexes, aged between 18 and <65 years. Glycemic status classification was performed according to the criteria of the American Diabetes Association (ADA). PLWHIV participants were recruited from the HCG-FAA HIV Unit; specifically, individuals receiving ART were included, with an undetectable viral load for at least one year, and a CD4^+^ T-cell count > 300 cells/μL. In total, 80 participants were included, and were distributed into five groups (16 per group) according to their glycemic status and the presence or absence of HIV infection, as follows: normoglycemic PLWHIV (PLWHIV), PLWHIV with prediabetes (PLWHIV+preT2D), PLWHIV with type 2 diabetes (PLWHIV+T2D), people without HIV with T2D (T2D), and a population control group (Control).

### 4.3. Monitoring of HIV Infection

The viral load and CD4^+^ T-cell count were determined at the State Public Health Laboratory of Guadalajara, in Guadalajara, Jalisco, Mexico. Viral load was quantified using an automated RT-PCR molecular diagnostic system, COBAS 5800 by Roche (Roche, Basel, Switzerland). CD4^+^ T-cell count was performed using the AQUIOS CL flow cytometer (Beckman Coulter, Brea, CA, USA).

### 4.4. Clinical, Sociodemographic, and Anthropometric Data and Sample Collection

After verification of the study eligibility criteria, physicians from the HCG-FAA HIV Unit conducted a routine clinical evaluation, with data recorded in the SMART medical platform, from which clinical and sociodemographic variables were subsequently accessed and analyzed.

At the Institute of Immunodeficiencies and HIV (InIVIH) of the University of Guadalajara, in Guadalajara, Jalisco, Mexico, anthropometric measurements were obtained and, after confirmation of an approximately 8 h fasting period, blood samples in tubes with K_2_EDTA and with clot activator were collected by venipuncture. After 10 min at room temperature, the tubes were centrifuged, aliquoted, and stored at −80 °C until analysis for quantification of inflammatory markers, mitochondrial dysfunction, and circulating miRNA, or, alternatively, sent to the Central Laboratory of HCG-FAA for hematological assessments, as well as glycemic and lipid profile determinations.

### 4.5. Hematological, Glycemic, and Lipid Profiles

One tube with K_2_EDTA and one with clot activator were transported to the Central Laboratory of HCG-FAA. Lipid and glycemic profiles were determined by colorimetric quantification (AU5800 autoanalyzer, Coulter Beckman, Brea, CA, USA) and by photometry (AU5800 autoanalyzer, Coulter Beckman, Brea, CA, USA), respectively.

### 4.6. Quantification of Inflammatory Markers and Mitochondrial Dysfunction

One tube with clot activator was centrifuged at 1800 rpm for 10 min, and the serum was aliquoted and stored at −80 °C until analysis. Levels of IL-6 (Cat. No. ab46042, Abcam, Cambridge, MA, USA) and hs-CRP (Cat. No. EIA-3954, DGR Instruments GmbH, Marburg, Germany), as well as IL-10 (Cat. No. BMS215-2), TNF-α (Cat. No. KAC1751), and GDF-15 (Cat. No. EHGDF15, this and the previous ones from Invitrogen, Carlsbad, CA, USA) were quantified in serum samples by ELISA assays following the manufacturer’s instructions. Optical densities (OD) were measured in a BioTek Synergy H1 microplate reader (BioTek, Winooski, VT, USA) at the specific wavelength indicated for each analyte. The OD obtained was transformed into concentrations (pg/mL or ng/mL) as per the manufacturer’s instructions for each kit used.

IL-8 was quantified by flow cytometry using the LEGENDplex™ Human Inflammation Panel 1 kit (Cat. No. 740809, BioLegend, Inc., San Diego, CA, USA). More than 2000 events per sample were considered in the reading performed on the Attune Acoustic Focusing Cytometer (Life Technologies, Carlsbad, CA, USA); data were analyzed using LEGENDplex™ QOGNIT virtual software (https://www.biolegend.com/en-us/immunoassays/legendplex/support/software, accessed on 1 November 2023; BioLegend, Inc., San Diego, CA, USA).

### 4.7. Extraction and Reverse Transcription of Circulating miRNAs

One tube with K_2_EDTA was centrifuged at 3000 rpm for 15 min at room temperature, using an acceleration setting of 3 and a brake setting of 2. Subsequently, plasma was carefully collected up to 1 cm above the buffy coat layer to avoid contamination with other plasma or cellular components. The plasma was then stored at −80 °C until analysis.

Total RNA was isolated from 200 μL of K_2_EDTA plasma using the miRNeasy Serum/Plasma Mini Kit (Cat. No. 217184, QIAGEN, Venlo, The Netherlands) according to the manufacturer’s specifications. RNA quality and purity were evaluated by spectrophotometry (Take3, Synergy H1, BioTek, Winooski, VT, USA). cDNA synthesis was performed from 20 ng of RNA using the miRCURY LNA RT kit from Qiagen (Cat. No. 339340, QIAGEN, Venlo, The Netherlands) according to manufacturer instructions.

### 4.8. Quantification of Circulating miRNAs—dPCR

Absolute quantification of miRNAs was performed by digital PCR (dPCR) using the QIAcuity System (Qiagen, Hilden, Germany). Quantification of miR-23a-3p was carried out using the QIAcuity EvaGreen PCR Kit (Cat. No. 250111; QIAGEN GmbH, Hilden, Germany) and a specific miRCURY LNA primer assay (Cat. No. YP00204772). Reactions were prepared in a final volume of 40 µL containing: 11 μL of EvaGreen Master Mix (3×), 2 μL of miRCURY LNA PCR primers (10×), 25 μL of RNAse-free water, and 2 μL of diluted (1:3) cDNA. Samples and negative controls were loaded into QIAcuity Nanoplate 26k nanoplates (Cat. No. 250031), sealed, and analyzed on the QIAcuity One instrument (QIAGEN GmbH, Hilden, Germany). Thermal cycling conditions consisted of 95 °C for 2 min, followed by 45 cycles at 95 °C for 15 s and 60 °C for 1 min. Data acquisition was performed in the green channel, and the results were analyzed using QIAcuity Software Suite 2.2.0.26 (Qiagen, Venlo, The Netherlands). miRNA expression levels were reported as absolute quantification (copies/µL) based on Poisson statistics.

### 4.9. Calculation of Inflammation and Insulin Resistance Indices

HOMA indices (HOMA2-IR, HOMA2-%β, HOMA2-%S) were calculated using the HOMA calculator version 2.2.3., provided by the Radcliffe Department of Medicine, University of Oxford.

Inflammation indices [14] as well as surrogate indices of IR [15] were calculated using their respective equations, which are presented below.

MLR=Monocytes(thousand/μL)/Lymphocytes(thousand/μL)

$$SIRI=\frac{Neutrophiles(thousand/\mu L)*Monocytes(thousand/\mu L)}{Lymphocytes(thousand/\mu L)}$$

$$AISI=\frac{Neutrophiles(thousand/\mu L)*Platelets(thousand/\mu L)*Monocytes(thousand/\mu L)}{Lymphocytes(thousand/\mu L)}$$

$$TyG=ln\frac{\left[Triglycerides(mg/dL)*FastingGlucose(mg/dL)\right]}{2}$$

$$METS-IR=\frac{ln[2*FastingGlucose(mg/dL)+Triglycerides(mg/dL)]*BMI}{ln[HDL-C(mg/dL)]}$$

### 4.10. In Silico Analysis

An in silico analysis was performed to identify and characterize the target genes regulated by miR-23a-3p. Interacting targets backed by robust experimental evidence were retrieved from the miRTarBase database (accessed in January 2026), filtering exclusively for interactions validated through stringent functional assays, including luciferase reporter assays, Western blot, and qPCR. To expand the regulatory landscape, computational target predictions were complemented using the TargetScan database based on seed-sequence complementarity and evolutionary conservation of the binding sites.

Subsequently, the validated and predicted interactions were integrated to construct a comprehensive regulatory network, which was visualized and structurally analyzed using Cytoscape software (Version 3.10.4). Within the network architecture, edges were distinctively mapped to reflect the level of supportive evidence: continuous lines were assigned to targets with experimental validation, whereas dashed lines represented the in silico predictive interactions. The interaction types were mapped as topological interactions without assuming directional mechanisms or direct post-transcriptional inhibition.

To mathematically validate the biological relevance of the interactome, an over-representation analysis (ORA) was executed using the STRING Enrichment application integrated into Cytoscape. The target gene set was analyzed against the whole Homo sapiens genome as the statistical background to compute both nominal *p*-values and False Discovery Rates (FDR, q-values). Multiple testing corrections were strictly adjusted using the Benjamini–Hochberg method, establishing a significance threshold of FDR < 0.05. Functional annotations and pathway mapping were comprehensively retrieved across the Kyoto Encyclopedia of Genes and Genomes (KEGG), Gene Ontology (GO) Biological Processes, and WikiPathways repositories to determine the coordinated biological axes regulated by miR-23a-3p.

### 4.11. Statistical Analysis

Population characterization was performed through descriptive analyses, using measures of central tendency (mean or median) and dispersion (standard deviation or interquartile range) for the quantitative variables, according to data distribution. Qualitative variables were expressed as frequencies and percentages. Data normality was evaluated using the Shapiro–Wilk test. Comparisons between groups were performed using the Kruskal–Wallis test, followed by Dunn’s post hoc analysis. For categorical variables, chi-square (χ^2^) tests or Fisher’s exact test were used, as appropriate.

Associations between continuous variables, including miR-23a-3p expression and inflammatory and metabolic biomarkers, were evaluated using Spearman correlation coefficients. To account for multiple testing, *p*-values derived from correlation analyses were adjusted using the Benjamini–Hochberg false discovery rate (FDR) correction. The discriminatory capacity of biomarkers was analyzed using ROC (Receiver Operating Characteristic) curves, and optimal cut-off points were determined using the Youden index. All statistical analyses were performed using IBM SPSS Statistics (version 29.0.2.0), GraphPad Prism (Version 10.6.1), and RStudio (Version 2026.01.1+403). A *p*-value < 0.05 was considered statistically significant.

## 5. Conclusions

This study provides novel evidence supporting an association between miR-23a-3p expression and HIV-associated prediabetes. The overexpression observed in PLWHIV with preT2D, together with its discriminative capacity for identifying this group, suggests that miR-23a-3p may represent a potential biomarker of early immunometabolic alterations in the context of HIV infection. Its association with IL-6 further supports a relationship with inflammatory and cellular stress-related processes. Additionally, in silico network and functional enrichment analyses identified pathways related to immune regulation, inflammation, and metabolic homeostasis, although these findings require experimental validation. Future longitudinal and functional studies are needed to confirm the biological and clinical relevance of miR-23a-3p in HIV-associated prediabetes.

## Figures and Tables

**Table 1:** Sociodemographic and anthropometric data.

	PLWHIV (*n* = 16)	PLWHIV+preT2D (*n* = 16)	PLWHIV+T2D (*n* = 16)	T2D (*n* = 16)	CONTROL (*n* = 16)	*p*-Value
Age (years)	38 ± 11	46 ± 10	48 ± 7	53 ± 9	43 ± 16	**0.008**
Gender						**0.006**
Male	12 (75)	16 (100)	14 (87.5)	9 (56.3)	8 (50)	-
Female	4 (25)	-	2 (12.5)	7 (43.7)	8 (50)	-
Weight (kg)	69.3 ± 9	74.5 ± 16	84.9 ± 17	80 ± 3	75.5 ± 16	**0.035**
Height (m)	1.71 (1.65–1.73)	1.79 (1.64–1.74)	1.71 (1.68–1.76)	1.69 (1.61–1.71)	1.67 (1.59–1.79)	0.639
BMI (kg/m^2^)	22.5 (22.4–26)	24.6 (20.5–30.2)	28.4 (25.1–32.7)	29.4 (26.4–31.6)	26.2 (24.3–28)	**0.009**
Waist-to-hip Ratio	0.87 (0.6–1)	0.88 (0.8–1.3)	0.96 (0.84–1)	0.93 (0.7–1)	0.86 (0.7–0.9)	0.010

Data are presented as mean ± standard deviation or median (interquartile range) according to data distribution. Overall group comparisons were performed using the Kruskal–Wallis test, and corresponding *p*-values are reported in the table. Dunn’s post hoc test was applied when the overall test was significant. Bold values indicate statistical significance (*p* < 0.05). Abbreviations: PLWHIV, people living with HIV; preT2D, prediabetes; T2D, type 2 diabetes.

**Table 2:** Clinical data of HIV infection.

	^1^ PLWHIV (*n* = 16)	^2^ PLWHIV+preT2D (*n* = 16)	^3^ PLWHIV+T2D (*n* = 16)	*p*-Value	Dunn-Adjusted *p*-Value
Dx time (Years)	6 ± 5	12 ± 8	10 ± 6	**0.009**	1 vs. 2 = 0.0116
Current ART				0.171	
Biktarvy^®^	16 (100)	15 (93.8)	15 (93.8)	-	
Atripla	-	1 (6.2)	-	-	
Truvada + ATV/r	-	-	1 (6.2)	-	NA
Time on ART (Years)	6 ± 6	10 ± 5	10 ± 6	**0.011**	1 vs. 2 = 0.0316 1 vs. 3 = 0.0274
Viral load (copies/mL)	31 (20–39)	20 (20–35)	31 (20–40)	0.281	NA
CD4^+^ T cells (cells/μL)	589 (427–705)	578 (426–636)	662 (414–946)	0.733	NA
Nadir CD4^+^ T cells (cells/μL)	299 (189–428)	218 (58–300)	512 (188–602)	0.111	NA
Ratio CD4^+^/CD8^+^	0.333 (0.175–0.621)	0.623 (0.089–5.15)	0.362 (0.18–4.94)	0.240	NA

Data are presented as mean ± standard deviation or median (interquartile range), depending on the data distribution. Categorical variables are expressed as percentages. Comparisons between groups were performed using the Kruskal–Wallis test (*p*-value) followed by Dunn’s post hoc test (Dunn-adjusted *p*-value). Bold values indicate statistical significance (*p* < 0.05). Superscript numbers (1–3) in the group columns indicate significant pairwise comparisons adjusted using Dunn’s post hoc test. Abbreviations: Dx, Diagnosis Time; ART, antiretroviral therapy; ATV/r, Atazanavir/ritonavir; PLWHIV, people living with HIV; preT2D, prediabetes; T2D, type 2 diabetes; NA, not applicable.

**Table 3:** Hematological, glycemic, and lipidic profiles.

Hematological Profile							
	^1^ PLWHIV (*n* = 16)	^2^ PLWHIV+ preT2D (*n* = 16)	^3^ PLWHIV+ T2D (*n* = 16)	^4^ T2D (*n* = 16)	^5^ CONTROL (*n* = 16)	*p*-Value	Dunn-Adjusted *p*-Value
Hemoglobin (g/dL)	15.3 ± 1.5	15.6 ± 0.81	15.5 ± 1.6	13.5 ± 1.7	15 ± 0.91	**<0.001**	1 vs. 4 = 0.0403 2 vs. 4 = 0.0028 3 vs. 4 = 0.0079
Platelets (miles/μL)	229 (140–305)	215 (127–263)	215 (142–410)	222 (27–304)	233 (163–330)	0.736	NA
Hematocrit (%)	46.1 (37.7–52)	47 (42–52)	47 (31–51)	46 (28–53)	44 (41–51)	0.198	NA
Leukocytes (%)	5.6 ± 1.4	6.7 ± 2.4	6.5 ± 1.8	6.5 ± 1.8	6.3 ± 1.5	0.389	NA
Lymphocytes (%)	34.1 ± 11	32.5 ± 11	38.9 ± 11	33.8 ± 8.5	32.1 ± 5.5	0.336	NA
Monocytes (%)	8.9 ± 2.2	7.9 ± 2.5	7.3 ± 1.9	5.9 ± 2.5	6.7 ± 2.7	**0.012**	1 vs. 4 = 0.0091
Neutrophils (%)	51 ± 12	56 ± 14	49 ± 13	59 ± 10	57 ± 8	00.133	NA
Glycemic and lipidic profiles							
Glucose (mg/dL)	83.5 (69–101)	85 (72–100)	138 (80–281)	148 (79–254)	82.5 (70–99)	**<0.001**	1 vs. 3 = 0.0012 1 vs. 4 = 0.0017 2 vs. 3 = 0.0064 2 vs. 4 = 0.0089 3 vs. 5 = 0.0001 4 vs. 5 = 0.0002
HbA1c (%)	5.4 ± 0.4	5.7 ± 0.3	8.3 ± 2.3	7.1 ± 1.7	5.7 ± 0.3	**<0.001**	1 vs. 3 < 0.0001 1 vs. 4 < 0.0001 2 vs. 3 = 0.0014 2 vs. 4 = 0.0175 2 vs. 5 = 0.0022 4 vs. 5 = 0.0251
Insulin (uU/mL)	11 (3–44)	10 (3–45)	18 (6–39)	26 (6–48)	9.5 (5–34)	**0.008**	1 vs. 4 = 0.0193
Urea (mg/dL)	27 (16–55)	31 (20–46)	33 (22–48)	29 (16–53)	32 (17–76)	0.253	NA
Creatinine (mg/dL)	0.83 ± 0.17	0.87 ± 0.15	0.8 ± 0.1	0.72 ± 0.14	0.80 ± 0.16	0.069	NA
Uric acid (mg/dL)	5.3 ± 1.1	5.7 ± 1.2	6.0 ± 1.4	4.3 ± 0.9	5.4 ± 1.4	**0.002**	2 vs. 4 = 0.0079 3 vs. 4 = 0.0004
Total cholesterol (mg/dL)	176 (116–368)	176 (123–266)	172 (127–250)	163 (101–252)	197 (112–260)	0.393	NA
Triglycerides (mg/dL)	111.5 (45–368)	105 (56–333)	180 (76–546)	144 (67–302)	121 (53–476)	0.100	NA
LDL-C (mg/dL)	108 ± 37	105 ± 27	99.5 ± 34	98.3 ± 36	107 ± 35	0.901	NA
HDL-C (mg/dL)	42 (27–66)	42.5 (27–67)	32 (26–45)	42.6 (24–58)	49 (24–114)	**<0.001**	1 vs. 3 = 0.0161 2 vs. 3 = 0.0357 3 vs. 5 = 0.0003
VLDL-C (mg/dL)	22 (9–74)	21 (11–67)	32.5 (15–60)	28.5 (15–60)	22.5 (10.6–95.2)	0.093	NA

Data are presented as mean ± standard deviation or median (interquartile range), depending on the data distribution. Categorical variables are expressed as percentages. Comparisons between groups were performed using the Kruskal–Wallis test (*p*-value) followed by Dunn’s post hoc test (Dunn-adjusted *p*-value). Bold values indicate statistical significance (*p* < 0.05). Superscript numbers (1–5) in the group columns indicate significant pairwise comparisons adjusted using Dunn’s post hoc test. Abbreviations: PLWHIV, people living with HIV; preT2D, prediabetes; T2D, type 2 diabetes; NA, not applicable.

**Table 4:** Inflammatory, systemic inflammation-related, metabolic stress, and insulin resistance (IR) biomarkers.

Inflammatory Biomarkers							
	^1^ PLWHIV (*n* = 16)	^2^ PLWHIV+ preT2D (*n* = 16)	^3^ PLWHIV+ T2D (*n* = 16)	^4^ T2D (*n* = 16)	^5^ CONTROL (*n* = 16)	*p*-Value	Dunn-Adjusted *p*-Value
IL-6 (pg/mL)	2 (0.20–4.10)	1.9 (0.15–5.92)	2.9 (0.68–16.7)	1.9 (0.10–11)	1.6 (0.35–5.34)	0.307	NA
IL-8 (pg/mL)	103.6 (4.8–211)	67.7 (35.7–354.8)	93 (28–184.4)	62.8 (17–164.4)	79 (14–175)	0.2548	NA
IL-10 (pg/mL)	2.5 (1–38)	1.6 (0.7–7.9)	3 (0.68–381)	2.9 (1–312)	2.6 (1.9–46)	**0.017**	2 vs. 5 = 0.0169
hs-CRP (mg/L)	2.5 (0.3–70)	1.8 (0.7–105)	18 (2–170)	4.5 (1–114)	2 (0.8–45)	**0.008**	1 vs. 4 = 0.00433 3 vs. 5 = 0.00095
TNF-α (pg/mL)	34.3 (24.3–53)	33 (21.9–42.9)	41.2 (29–54.7)	33.5 (25.9–50.4)	30.2 (23.5–47.9)	**0.0074**	2 vs. 3 = 0.0128 3 vs. 5 = 0.0139
Systemic Inflammation-Related and Metabolic Stress Biomarkers							
SIRI	0.78 (0.33–2.55)	0.82 (0.28–2.8)	0.68 (0.11–1.2)	0.49 (0.26–2.2)	0.74 (0.29–1.4)	0.1308	NA
AISI	194.8 (62.6–629.6)	183.7 (61.6–736)	139.2 (16.9–384.7)	89.7 (8.6–667.8)	170.9 (57.8–451)	0.1655	NA
MLR	0.017 ± 0.011	0.018 ± 0.010	0.013 ± 0.005	0.0107 ± 0.008	0.012 ± 0.004	**0.0325**	2 vs. 4 = 0.0407
GDF-15 (pg/mL)	386.8 (79–892)	649 (317–1215)	729 (437–1510)	724 (336–1531)	340 (216–1033)	**<0.0001**	1 vs. 2 = 0.0433 1 vs. 3 = 0.0011 1 vs. 4 = 0.0250 2 vs. 5 = 0.0164 3 vs. 5 = 0.0003 4 vs. 5 = 0.0090
	Surrogate Insulin Resistance Indices						
HOMA2-IR	1.4 (0.3–5–5)	1.2 (0.4–5.3)	2.6 (0.8–5.2)	3.9 (0.7–7)	1.2 (0.6–4.2)	**0.0014**	1 vs. 4 = 0.0080 2 vs. 4 = 0.0352 4 vs. 5 = 0.0459
HOMA2-%β	123.9 (43.5–282)	120.2 (40.9–384)	60 (10.4–274)	101.6 (19–279)	146 (73–332)	**0.039**	3 vs. 5 = 0.067
HOMA2-%S	71 (18–269)	82 (5.2–247)	38 (19–118)	25 (4–132)	83 (23–161)	**0.0014**	1 vs. 4 = 0.0069 2 vs. 4 = 0.0369 4 vs. 5 = 0.0431
TyG	8.4 (7.5–9.7)	8.4 (7.7–9.6)	9.5 (8.1–11)	9.3 (8.2–10.2)	8.5 (7.7–9.8)	**0.0002**	1 vs. 3 = 0.0049 1 vs. 4 = 0.0246 2 vs. 3 = 0.0093 2 vs. 4 = 0.0433
METS-IR	37.3 (26.5–48.5)	38.8 (24.7–57.3)	52.1 (35.2–70.4)	47.2 (35–62.5)	38.9 (23–55)	**<** **0.** **0001**	1 vs. 3 < 0.0001 1 vs. 4 = 0.0054 2 vs. 3 = 0.0002 2 vs. 4 = 0.0268 3 vs. 5 = 0.0004 4 vs. 5 = 0.0431

Data are presented as mean ± standard deviation or median (interquartile range), depending on the data distribution. Categorical variables are expressed as percentages. Comparisons between groups were performed using the ANOVA or Kruskal–Wallis test (*p*-value) followed by Tukey’s or Dunn’s post hoc test (Dunn-adjusted *p*-value). Bold values indicate statistical significance (*p* < 0.05). Superscript numbers (1–5) in the group columns indicate significant pairwise comparisons adjusted using Dunn’s post hoc test. Abbreviations: PLWHIV, people living with HIV; preT2D, prediabetes; T2D, type 2 diabetes; NA, not applicable.

**Table 5:** Diagnostic performance and optimal cut-off points of the biomarkers evaluated in the study cohort.

Biomarker	AUC (IC 95%)	*p*-Value	Cut-Off	Se (%)	Sp (%)
PLWHIV vs. PLWHIV+preT2D					
miR-23a-3p (copies/μL)	0.80 (0.6576–0.9591)	0.0034	>185.9	87	66
GDF-15 (pg/mL)	0.83 (0.6856–0.9745)	0.0014	>590.6	68	93
HbA1c (%)	0.76 (0.5860–0.9473)	0.0166	>5.5	73	69
METS-IR	0.53 (0.3279–0.7424)	0.7345	<37.0	50	93
PLWHIV vs. PLWHIV+T2D					
miR-23a-3p (copies/μL)	0.70 (0.5143–0.8941)	0.05	>160.3	81	66
GDF-15 (pg/mL)	0.89 (0.6856–0.9745)	0.0002	>581.5	75	93
Glucose (mg/dL)	0.89 (0.7778–1.000)	0.0001	>103.5	75	100
HbA1c (%)	0.96 (0.9132–1.000)	<0.0001	>6.1	87	100
HOMA2-IR	0.78 (0.6207–0.9496)	0.0059	>1.66	75	75
TyG	0.85 (0.7190–0.9842)	0.0007	>8.745	87	75
METS-IR	0.89 (0.7835–1.000)	0.0002	>45	81	62
CONTROL vs. T2D					
miR-23a-3p (copies/μL)	0.67 (0.4929–0.8665)	0.083	<279.7	75	56
GDF-15 (pg/mL)	0.83 (0.6995–0.9802)	0.001	>393.5	87	68
Glucose (mg/dL)	0.89 (0.7680–1.000)	0.0001	>101	81	100
HbA1c (%)	0.85 (0.7099–0.9943)	0.005	>6.4	68	100
HOMA2-IR	0.79 (0.6268–0.9566)	0.0057	>2.125	80	75
TyG	0.74 (0.5797–0.9163)	0.0167	>9.185	56	81
METS-IR	0.78 (0.6170–0.9455)	0.0067	>42.2	87	68

Data are presented as mean ± standard deviation or median (interquartile range), as appropriate. AUC: area under the receiver operating characteristic curve; 95% CI: 95% confidence interval; Se: sensitivity; Sp: specificity. Optimal cut-off values were determined using the Youden index. Comparisons were performed for the following scenarios: PLWHIV vs. PLWHIV+preT2D, PLWHIV vs. PLWHIV+T2D, and Control vs. T2D. *p* < 0.05 was considered statistically significant. PLWHIV, people living with HIV; PLWHIV+preT2D, people living with HIV + prediabetes; PLWHIV+T2D, people living with HIV + diabetes; T2D, type 2 diabetes.

**Table 6:** miR-23a-3p logistic regression analysis.

	OR	IC 95%	*p*-Value
Model 1	7.22	1.57–42.27	0.0163
Model 2	4.99	0.89–33.30	0.074
Model 3	14.67	2.62–168.9	0.0009

OR: odds ratio; 95% CI: 95% confidence interval. Model 1: unadjusted logistic regression using miR-23a-3p, categorized according to the optimal cut-off (>185.9 copies/µL). Model 2: logistic regression adjusted for age and body mass index (BMI) using categorized miR-23a-3p. Model 3: logistic regression using miR-23a-3p as a continuous variable (log10-transformed expression levels). Outcome variable: preT2D in PLWHIV. *p* < 0.05 was considered statistically significant.

## Data Availability

The data presented in this study are available on request from the corresponding authors. The data are not publicly available due to ethical and privacy restrictions related to participant confidentiality.

## Supplementary Materials

The following supporting information can be downloaded at: https://www.mdpi.com/article/10.3390/ijms27135658/s1.

## Abbreviations

The following abbreviations are used in this manuscript:

ADA	American Diabetes Association
AISI	Aggregate Index of Systemic Inflammation
AKT	Protein kinase B
APAF1	Apoptotic protease-activating factor 1
AUC	Area under the curve
ART	Antiretroviral therapy
BMI	Body mass index
CD4^+^	Cluster of differentiation 4 positive T lymphocytes
CD8^+^	Cluster of differentiation 8 positive T lymphocytes
CI	Confidence interval
dPCR	Digital polymerase chain reaction
ELISA	Enzyme-linked immunosorbent assay
GDF-15	Growth differentiation factor 15
HbA1c	Glycated hemoglobin
HCG-FAA	Hospital Civil de Guadalajara “Fray Antonio Alcalde”
HDL-C	High-density lipoprotein cholesterol
HOMA2-B	Homeostatic Model Assessment 2 of beta-cell function
HOMA2-IR	Homeostatic Model Assessment 2 of insulin resistance
HOMA2-S	Homeostatic Model Assessment 2 of insulin sensitivity
hs-CRP	High-sensitivity C-reactive protein
HIV	Human immunodeficiency virus
IL-6	Interleukin 6
IL-8	Interleukin 8
IL-10	Interleukin 10
IL6R	Interleukin 6 receptor
InIVIH	Institute of Immunodeficiencies and HIV
IRF1	Interferon regulatory factor 1
JAK/STAT	Janus kinase/Signal transducer and activator of transcription pathway
KLF3	Kruppel-like factor 3
LDL-C	Low-density lipoprotein cholesterol
METS-IR	Metabolic Score for Insulin Resistance
miRNA	MicroRNA
miR-23a-3p	MicroRNA-23a-3p
MLR	Monocyte-to-lymphocyte ratio
NF-κB	Nuclear factor kappa B
OD	Optical density
OR	Odds ratio
PCR	Polymerase chain reaction
PDE7A	Phosphodiesterase 7A
PI3K/AKT	Phosphoinositide 3-kinase/AKT signaling pathway
PLWHIV	People living with HIV
preT2D	Prediabetes
PRKCA	Protein kinase C alpha
PTEN	Phosphatase and tensin homolog
qPCR	Quantitative polymerase chain reaction
ROC	Receiver operating characteristic
RNA	Ribonucleic acid
rpm	Revolutions per minute
S6K	Ribosomal protein S6 kinase
Se	Sensitivity
SIRI	Systemic Inflammation Response Index
Sp	Specificity
SPRY2	Sprouty RTK signaling antagonist 2
T2D	Type 2 diabetes
TNF-α	Tumor necrosis factor alpha
TNFAIP3	TNF alpha-induced protein 3
TRAF5	TNF receptor-associated factor 5
TyG	Triglyceride-glucose index
µL	Microliter
VLDL	Very low-density lipoprotein
