# Supplementary material for: miR-23a-3p as a Biomarker Associated with Prediabetes in People Living with HIV: An Integrative Analysis of Inflammatory, Metabolic, and Insulin Resistance Signatures
Source: Int J Mol Sci. 2026 Jun 23;27(13):5658. doi: 10.3390/ijms27135658 (PMC13361635; doi:10.3390/ijms27135658)
Supplement: Supplementary file 1 [file ijms-27-05658-s001.zip › Supplementary Figure 1.pdf]

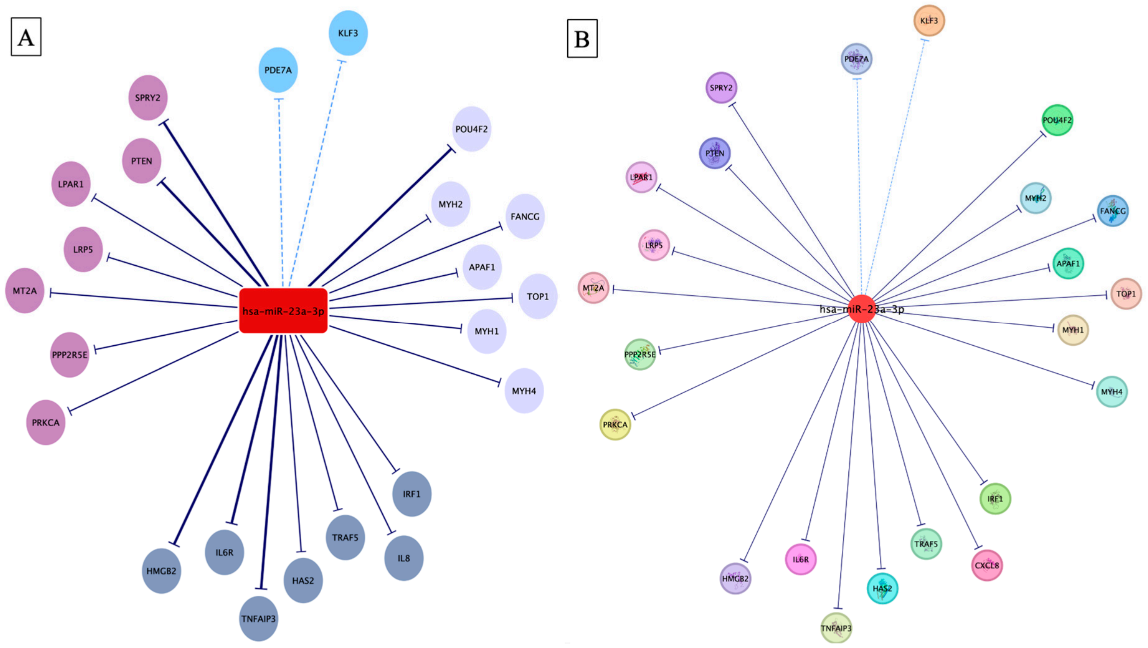

Supplementary Figure S1. Topological architecture and functional clustering of the hsa-miR-23a-3p interaction network. (A) Decentralized hub-and-spoke layout of the network mapping hsa-miR-23a-3p and its 25 experimentally validated (miRTarBase-supported) or in silico predicted target genes (TargetScan-based). Experimentally validated interactions are represented by solid edges, whereas predicted interactions are represented by dashed edges. (B) Functional clustering representation of the same interactome, optimized with a solid rectangular hub for hsa-miR-23a-3p.
