# Supplementary material for: miR-23a-3p as a Biomarker Associated with Prediabetes in People Living with HIV: An Integrative Analysis of Inflammatory, Metabolic, and Insulin Resistance Signatures
Source: Int J Mol Sci. 2026 Jun 23;27(13):5658. doi: 10.3390/ijms27135658 (PMC13361635; doi:10.3390/ijms27135658)
Supplement: Supplementary file 1 [file ijms-27-05658-s001.zip › Supplementary Table 1.pdf]

Supplementary Table S1. Functional enrichment analysis of gene targets regulated by hsa-miR-23a-3p

Category ID	Biological Term	Count	Genes	FDR	p value
**KEGG Pathways**					
hsa05200	Pathways in cancer	8	TRAF5, LRP5, CXCL8, IL6R, PTEN, LPAR1, PRKCA, APAF1	<0.0001	<0.0001
hsa04151	PI3K-Akt signaling pathway	5	PPP2R5E, IL6R, PTEN, LPAR1, PRKCA	<0.001	<0.01
hsa04064	NF-kappa B signaling pathway	3	TRAF5, CXCL8, TNFAIP3	<0.001	<0.05
hsa04668	TNF signaling pathway	3	IRF1, TRAF5, TNFAIP3	<0.001	<0.05
**GO Biological Process**					
GO:0012501	Programmed cell death	11	IRF1, TRAF5, POU4F2, LRP5, HMGB2, TOP1, IL6R, PTEN, PRKCA, APAF1, TNFAIP3	<0.0001	<0.001
GO:0006915	Apoptotic process	10	IRF1, TRAF5, POU4F2, LRP5, HMGB2, IL6R, PTEN, PRKCA, APAF1, TNFAIP3	<0.0001	<0.001
GO:0034097	Response to cytokine	9	MT2A, IRF1, TRAF5, POU4F2, CXCL8, HAS2, IL6R, SPRY2, PRKCA	<0.0001	<0.001
GO:0071345	Cellular response to cytokine stimulus	8	MT2A, IRF1, TRAF5, POU4F2, CXCL8, HAS2, IL6R, SPRY2	<0.0001	<0.01
**WikiPathways and STRING Clusters**					
WP4754	IL-18 signaling pathway	5	IRF1, CXCL8, PTEN, PRKCA, TNFAIP3	<0.001	<0.05
CL_22629	Myosin II Complex	3	MYH1, MYH4, MYH2	<0.0001	<0.01

Nominal p-values and false discovery rates (FDR, q-value) are expressed as statistical significance thresholds after correction for multiple comparisons using the Benjamini-Hochberg method. Overrepresentation analysis was performed using the STRING Enrichment tool within the Cytoscape v3.10.4 platform, with the complete Homo sapiens genome as the statistical background.
