# Supplementary figures and images for: miR-23a-3p as a Biomarker Associated with Prediabetes in People Living with HIV: An Integrative Analysis of Inflammatory, Metabolic, and Insulin Resistance Signatures
Source: Int J Mol Sci. 2026 Jun 23;27(13):5658. doi: 10.3390/ijms27135658 (PMC13361635; doi:10.3390/ijms27135658)

A

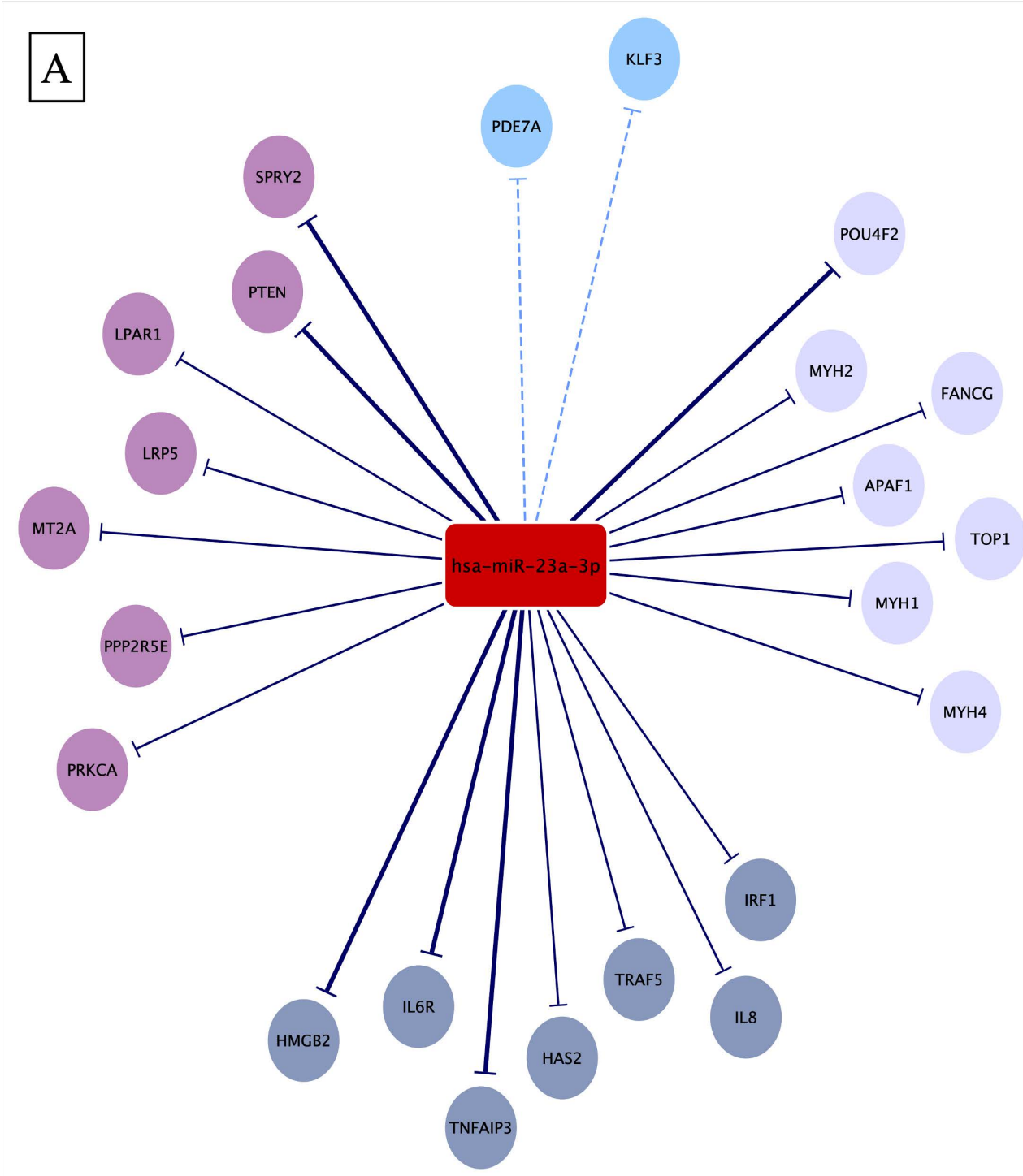

B

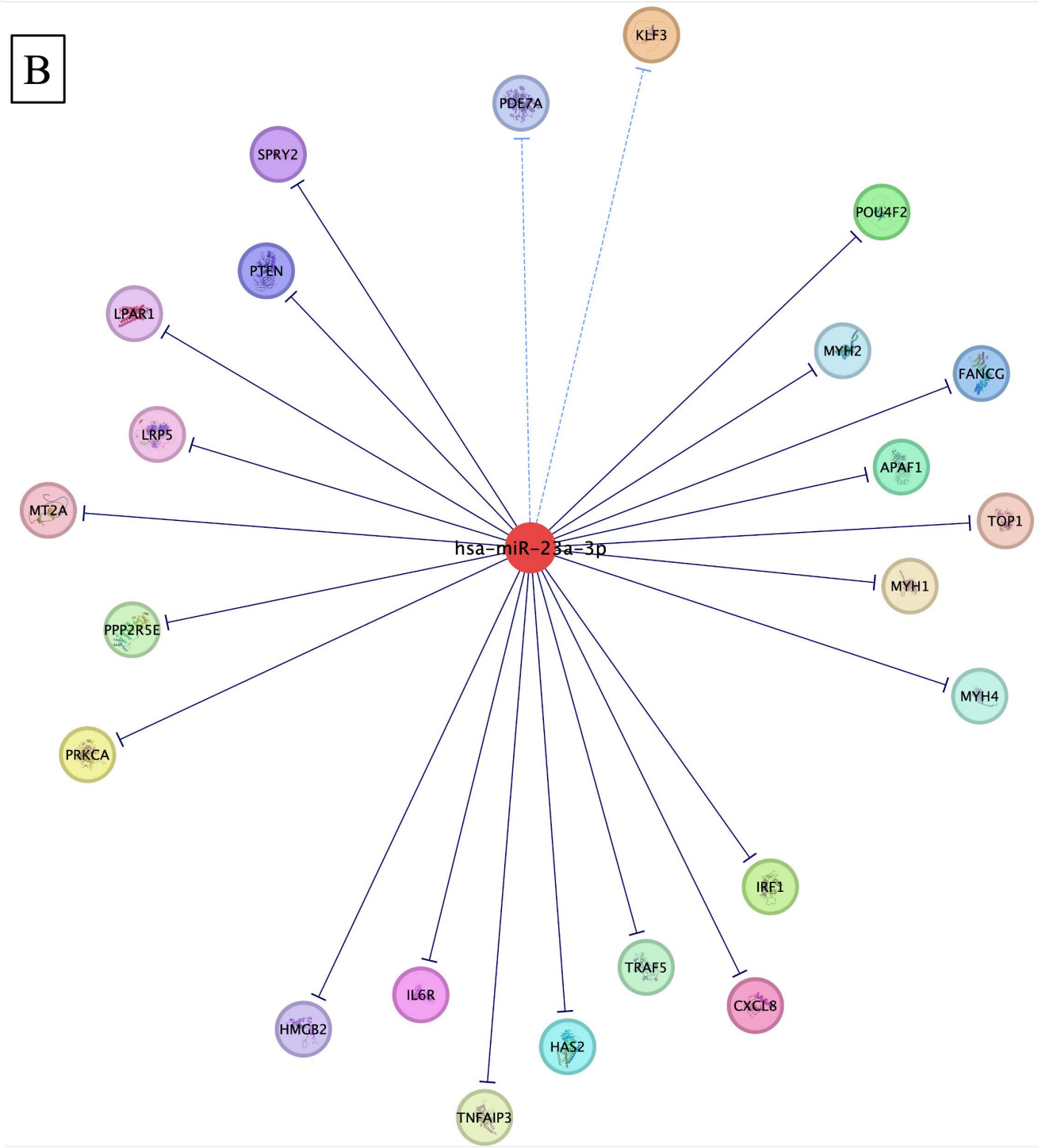

Supplement: Supplementary file 1 [file ijms-27-05658-s001.zip › FigureS1.pdf]
